# Longitudinal Health-Related Quality of Life Study among Cervical Cancer Patients Treated with Radiotherapy

**DOI:** 10.3390/jcm10020226

**Published:** 2021-01-10

**Authors:** Małgorzata Pasek, Lilia Suchocka, Grażyna Osuch-Pęcak, Konrad Muzykiewicz, Ewa Iwańska, Helena Kaducakowa, Anna Goździalska, Magdalena Goździalska

**Affiliations:** 1 Department of Nursing, Faculty of Health, University of Applied Sciences in Tarnow, 33-100 Tarnow, Poland; 2 Faculty of Education and Psychology, Jan Kochanowski University of Kielce, 25-029 Kielce, Poland; liliasuchocka@ibnps.eu; 3 National Oncology Institute Maria Sklodowska Curie-National Research Institute, Hospital of the National Institute of Oncology—Krakow Branch Poland, 31-115 Krakow, Poland; grosp@interia.pl (G.O.-P.); konrad.muzykiewicz@gmail.com (K.M.); grosp@poczta.onet.pl (E.I.); 4 Department of Nursing, Faculty of Health, Catholic University in Ruzomberok, 034 01 Ružomberok, Slovakia; helena.kaducakova@ku.sk; 5 Faculty of Health and Medical Studies, A.F. Modrzewski Krakow University, 30-705 Krakow, Poland; anna.gozdzialska@gmail.com; 6 Student’s Science Society of the Department of Infectious and Tropical Diseases and Hepatology, Medical University of Warsaw, 02-091 Warsaw, Poland; magda.gozdzialska@gmail.com

**Keywords:** quality of life, radiotherapy, cervical cancer, longitudinal study

## Abstract

Quality of life studies in medicine, particularly in oncology, have become a basic tool used to assess patient’s performance in different types of cancer and different modalities of treatment. The aim of this study was a subjective evaluation of the quality of life in cervical cancer patients treated with radiotherapy. The study has a longitudinal character and comprises four stages: before treatment, at the end of treatment, 5 months and 5 years after treatment. Standardized questionnaires such as EORTC QoL C30, HADS (European Organisation for Research and Treatment of Cancer Quality of life C30 Hospital Anxiety and Depression Scale) and the authors demographic–clinical assessment survey were the study tools. Physical functioning was assessed as the highest before treatment and depreciated to the lowest value 5 years after treatment. Emotional functioning was the lowest before treatment and then decreased again in the fourth stage of the assessment. Global quality of life was the lowest in the fourth stage of the study. Memory and concentration were fairly good at every stage of the study, with the highest score at the end of the treatment. At stages 3 and 4, the respondent’s social functioning was the best, followed by the ability to fulfil their social role. General health and quality of life were assessed by the respondents on a level slightly above average, though five years after treatment the score was slightly below average.

## 1. Introduction

Quality of life (QOL) studies in medicine, particularly in oncology, have become a basic tool used to assess patient’s performance in different types of cancer and different modalities of treatment. It seems that the therapeutic priority should not only be treating the cancer, but also reaching such a state that the patient feels that life is worth living. Research shows that, despite overall health improvement in the world, cervical cancer, as well as its treatment, negatively affects quality of life in all of its aspects [1]. Furthermore, patients who received multimodal treatment, radiochemotherapy, experienced more problems indicated in individual subscales, as well as in overall quality of life, than patients who underwent only surgery [2]. In comparison to operative treatment and chemotherapy, radiotherapy significantly lowers the quality of life. That was also confirmed in Ossan’s research [3], while Pfaendler stated that long-term QOL was strongly related to treatment modality, especially among patients that underwent extensive surgery or radiotherapy, who experienced persistent bladder, bowel and sexual disfunction [4].

Purposefulness of QOL studies in cervical cancer patients is further confirmed by epidemiological research in Poland, where more than 40% of patients are diagnosed with stage III or higher. Only about 27% of Polish women have cervical cytology performed regularly. Poland has one of the highest cancer mortality and morbidity indexes in Europe [5].

## 2. Experimental Section

The goal of this project was to assess the quality of life and its variables among cervical cancer patients treated with radiotherapy. The results are meant to be used to work out patient care recommendations and might be an important element in the individualized medical approach decision-making process in cancer treatment.

A diagnostic pool method was used in the study—a questionnaire technique. The study tools were the authors demographic–clinical assessment questionnaire as well as the standardized: QLQ 30 (Quality of life 30) [6] and HADS (Hospital Anxiety and Depression Scale) for anxiety and depression assessment. The detailed part (questionnaire CX24) was not used in the study because the first three of its parts took place when there was no access to a validated Polish translation [7].

In the interpretation of the functioning results in each dimension, a higher score indicates better QOL, whereas in the case of symptoms, a higher score (more frequent incidence) indicates a worse outcome.

The project received permission from the bioethical committee, acting in the National Institute of Oncology—Krakow Branch. Participation was voluntary. Confidentiality and good research practice rules were kept during all aspects of the study.

Descriptive statistics (mean, median, standard deviation, minimal and maximal value, relative and absolute value) were used in the data analysis. A Mann–Whitney test was used to assess the difference between qualitative and quantitative variables. *p* < 0.05 was set as the level of statistical significance. Calculations were made in the SPSS 20 program (IBM SPSS Statistics, USA).

The following were the inclusion criteria: diagnosis of cervical cancer, radiotherapy qualification, in-patient treatment, written consent to participate in the study, exclusion of mental illness. The exclusion criteria were: gynecological cancer organ other than cervical, inability to fill the questionnaire. 

**Figure 1:**
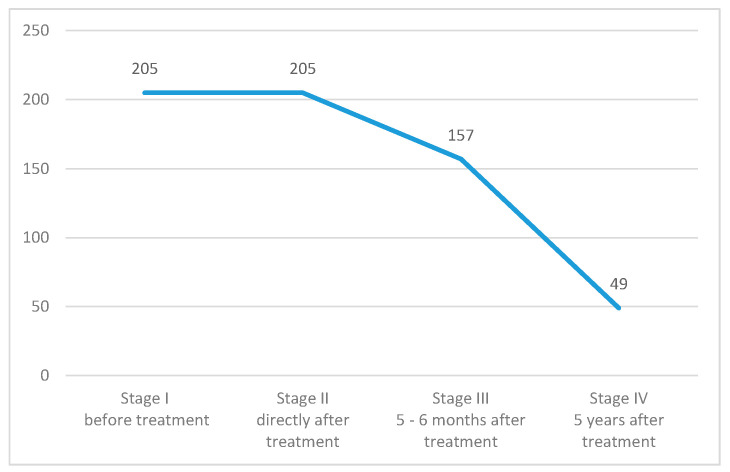
Number of women taking part in each stage of the study.

The research project was prospective, comprised of four stages, during which the respondents were asked to fill out a questionnaire. The first stage took place in the hospital before the start of radical radiotherapy, the second stage took place directly after the end of the treatment, before discharge. The third and fourth stages were correspondence questionnaires, in accordance with the patient’s prior consent. The patients were recruited during a hospital stay at a gynecologic oncology ward, where they were qualified to radical radiotherapy, meaning the treatment was with the intention to cure. Long-term studies, especially among cancer patients, carry a high risk of subject dropout. A group of 49 patients took part in all four parts of the study and underlie the statistical analysis (Figure 1). Women were recruited in five leading cancer centers in Poland (Krakow, Warszawa, Lublin, Gliwice, Kielce). The bioethical basis of our study was that the subject could drop out at any stage without giving a reason, and with no influence on the subsequent treatment.

Women who took part in the study were mostly married, of secondary or professional education, for whom job or benefit was the main income (Table 1). The mean age of the respondent was 53.37 years, in the range of 29–75 years.

The clinical characteristic of the study group is shown in Table 2, which indicates that the most common diagnosis was planoepitheliale FIGO stage IIIB cervical cancer. Most patients received 25–27 sessions of radiotherapy with a total dose of 48–50 Gy. In most of the patients, the treatment was combined with cisplatin-based chemotherapy. Only 27% of the women in the study group underwent surgery related to the cervical cancer diagnosis (Table 2).

## 3. Results

Women who participated in the study subjectively estimated their quality of life and overall health on each of the stages. For both variables, the mean value was above four, on a scale from 1 to 7, with a tendency to reach the lowest values at the last stage, five years after completion of radiotherapy (Table 3). After standardization of the results, overall quality of life was rated by the subjects as the lowest in the fourth stage of the study, at 58.78 (Table 3 and Table 4).

The results of the study show that physical functioning was assessed as best before the treatment started and reached its lowest five years after treatment. 

The emotional functioning was the worst of all stages before treatment and lowered again at stage IV compared to earlier stages.

Memory and concentration were assessed as relatively good at all stages, reaching the highest value of all subscales directly after radiotherapy.

At stages III and IV, the respondents pointed out social functioning, followed by the ability to fulfil their social roles as best. 

The lowest values at every stage of the study, indicating the biggest problem for the women in the study group, were related to financial difficulties as a consequence of illness (Table 5).

Our study has shown that the biggest difficulties at all of the stages were related to fatigue and insomnia (Rank 1 and 2). The fact that the women point at pain as present not only before treatment but also 5–6 months and 5 years after radiotherapy was worrying. 

At stage four of the study, symptoms such as pain, dyspnea, diarrhea, fatigue and insomnia worsened in comparison to their intensity at earlier stages (Table 6). 

The results of the study show that the level of statistical significance below 0.001 occurred between stages for physical functioning, role functioning and somatic symptoms, such as nausea and vomiting, loss of appetite and diarrhea (Table 7). Table 8 shows levels of statistical significance between each stage of the study. 

The subject of our study was also the examination of anxiety as a state and the occurrence of depression. Obtained results allowed the assertion that in the study group depression did not occur and the level of anxiety before treatment and 5 years after its completion was borderline. Directly after radiotherapy, and 5–6 months later, the mean level of anxiety was between normal (0–7) and borderline (8–10) (Table 9).

## 4. Discussion

Cervical cancer is the fourth most common cause of death due to cancer among women in Poland. Thanks to systematically introduced prophylactic, diagnostic and therapeutic standards, the morbidity index dropped from third place in year 1999 to eighth in 2017 [5]. The downturn in morbidity qualifies the disease as chronic. It implicates a change in therapy, where health-related quality of life becomes an important factor, which is the subject of this study. In our innovative research, we have conducted a longitudinal quality of life study among cervical cancer patients. Conducting a longitudinal QOL study in oncology carries a high risk of failure. On one hand, it concerns the dependence of the observation on the patient’s decision to participate; on the other hand, the patient’s death is a major drop-out factor. That is why there is paucity of these kinds of reports in the literature.

Despite those difficulties, we managed to conduct the project in five major Polish cancer centers. In total, 205 women were qualified and took part in the first two stages. Of which, 157 respondents took part in the third stage, which took place 5–6 months after treatment. Only 49 participants lasted until the fourth stage which was 5 years after treatment. Subjects that participated in all stages of the study were the basis for statistical analysis. A limitation of this study was the lack of a possibility to determine the cause of dropout, whether it was death or a decision to quit.

The participants of our study constitute a representative group, which is a strong aspect of the project. The most common histopathological diagnosis was ca. planoepitheliale (nearly 90%). The stage was IIA–IIIb among 74% of the respondents, which confirms that the treatment was in accordance with Polish recommendations. Cancer stage was similar in the Bashir study group [8]. Nearly 72% of the participants had combined treatment, which was radiotherapy with weekly cisplatin. Due to the size to the study group, we decided not to conduct a stratified analysis.

The mean age of the participant was 53 years, which is in agreement with Polish epidemiological data. The smallest group was women with higher education (10%). The vast majority of the respondents lived in relationships (married 73%, followed by widows, divorced and informal). This may explain a relatively high assessment of social functioning on all stages of the study. The source of income for most participants was either benefit, pension or welfare (together, nearly 49%) and for 30%—work. This might be the cause of reported financial difficulties. One of the most common causes of admission to the hospital during radiotherapy was the distance to the cancer center. This is reflected by the results of our study in which more than 80% of the respondents lived over 45 km away from the treatment facility. 

Women in our study rated their overall health and QOL on a level slightly above average. In the first stage, this assessment was slightly lower (60.71) than in stages two and three where the value exceeded 62. This might be explained by the fear of hospital stay and cancer treatment, which finds confirmation in emotional functioning analysis showing the lowest scores before treatment [9]. However, the opinions on QOL gave by respondents at the fourth stage of the study drew special attention, where the level (57.8) was the lowest of all stages. This finding did not confirm the results of the research by Dos Santos et al., which pointed out a relation between better QOL and longer time from treatment, though in the case of radiotherapy, the QOL was estimated as the lowest [10]. Schmidt et al. also indicated that, after five years, health and QOL were better compared to the state before treatment [11]. Grion, in his research, stated that higher family income, longer school education and not smoking were positively related to quality of life [12]. 

The low estimation of quality of life by women in our study might be caused by fatigue, insomnia and pain which worsened five years after treatment, compared to 6 months after it was completed. Apart from that, the respondents suffered from diarrhea, which in the literature is described as a complication of radiotherapy that becomes a chronic state after the acute phase ends and might not only cause physical disfunction but also problems in the social life. Research by Bjelic-Radisic et al. has shown that cervical cancer survivors assessed their QOL as good. Nevertheless, the result was negatively correlated with symptoms such as short breath, loss of appetite, nausea and vomiting, sleep disorders, peripheral neuropathy, and menopausal. There was also a positive relation between quality of life and economic status, as well as social functioning [13]. 

In our study, emotional functioning was best rated directly after treatment (60.37, above average). The values were low before the first (where it was the lowest) as well as in third and fourth stage. Fleming et al. indicated that the results concerning emotional functioning showed significant worsening that persisted after 6 weeks (*p* = 0.004), 6 months (*p* = 0.007), 1 year (*p* = 0.001), 2 years (*p* = 0.002) and 4 years (0 = 0.03) [8]. According to Bae et al., an additional cause of worse QOL assessment was sexual function, which was negatively correlated with mental status [14]. Research by Lee el at., on the other hand, showed that sexuality was not impaired in women after cervical cancer treatment who did not have symptoms of the disease and engaged in sexual activity, compared to healthy women [15].

Illness-related financial difficulties have been reported by women in our study, even though there was no statistical significance between stages. Huang et al. reported that, among cervical cancer survivors, only household income was related to quality of life [16].

The level of anxiety among respondents was borderline, depression did not occur. This is similar to Ferrandina’s results [17]. A thorough analysis of emotional disfunction was not the goal of our study, it was to point out a tendency or occurrence of anxiety or depression.

Demographic and clinical variables did not present statistically significant influence on somatic and functional subscales, as well as QOL in our own studies, which is confirmed by research by Pasek et al. [18]. Sungh et al., however, found that the cancer stage is an independent variable influencing quality of life in multivariate analysis [19]. Rahman et al. have shown that young women and people with higher level of education had better QOL. Stage and type of cancer affected overall QOL slightly, but respondents in early stages with well-differentiated diseases had better cancer-related quality of life [20]. 

Based on the research results, one can state that values of functional scales, excluding social sphere, have shown a worsening trend in QOL, statistically significant in relation to physical functioning between stages I and II, and I and IV, and too, in relation to role functioning between stages I and II, and II and III. Bashir et al. obtained similar results in regard to QLQ-30; mean results in all domains showed worsening in quality of life in comparison to initial status. This effect was more pronounced in emotional and social functioning. In relation to overall health and the EORTC QLQ CX-24 (European Organisation for Research and Treatment of Cancer Quality of life) questionnaire, all items have also showed worsening in quality of life compared to initial status [9].

Moreover, Prasogvej et al. found that cervical cancer survivors treated with radiotherapy had better results in emotional, social and overall health than the control group. Those patients had lower levels of appetite loss, fatigue and financial problems. On the other hand, patients treated with chemoradiotherapy suffered from more pain than the control group. All cervical cancer survivors had worse scores in regard to physical fitness than the control group [21]. 

The four-stage radiotherapy-treated cervical cancer patient QOL study allowed a unique analysis of changes in values of functional and somatic subscales as well as in anxiety and depression, occurring during care and treatment. Values of most of the variables were lower, especially in stage IV, 5 years after treatment, despite, as it may seem, significant improvement 5 months after treatment. Our results indicate a necessity of therapeutic intervention by a psycho-oncologist or a nurse, whose action would be aimed at individual effect after the oncologic treatment ends. Cancer, when it becomes a chronic disease, requires a more careful look and care over patients who live long after treatment, which is implied by the results of our research in a representative group of women. Research studies tend to assess that radiotherapy has a more negative influence on QOL compared to chemotherapy and operative treatment. 

The authors, in agreement with Pfaendler, postulate a focus on alleviation of long-term side effects, apart from systematic work on improving treatment results and survival. Providing supportive care and assessment of its effects might lower the occurrence and intensity of distant consequences of cervical cancer treatment, which might then improve quality of life and care [4]. 

Furthermore, inclusion of QOL monitoring into individualized patient care, in conjunction with proper interventions of the therapeutic team, might contribute to elaboration and actualization of evidence-based standards of care [1]. 

Cooperation between cancer centers and general practice and continuity of care may increase total health-related QOL assessment, as well as all the sub-scales. The fact that the patient feels she can get professional care at the right time (doctors, nurses, psychologists, physiotherapists, dietitians, social workers, etc.) makes her feel safe and improves QOL at each stage of cancer treatment.

Curiosity and the possibility of using the results to create and update standards of evidence-based medicine have outweighed the risks, and, hence, a project of subjective health-related QOL assessment among cervical cancer patients was conducted. Health status assessment and recognition of problems and needs may be, thanks to this study, based on empirical results. Our research may strengthen intuitive treatment and facilitate evidence-based practice.

QOL assessment using a standardized questionnaire might be included in routine follow-up in cancer patients. This would increase the size of the study group and bind the assessment result to a clinical setting. Such observation might facilitate sustaining higher quality of life and could also make thorough observation of anti-cancer treatment easier. Confidentiality and anonymity would have to be considered.

## 5. Conclusions

Based on the results of our research, the following conclusions have been drawn.

General health and quality of life among cervical cancer patients were assessed by the respondents on a level slightly above average, though five years after treatment the score was slightly below average, which is unique to our study.

Physical, role, social and emotional functioning differed significantly between stages.

Insomnia, fatigue and pain were the most commonly reported symptoms by the respondents (though the relation between stages was statistically insignificant).

## Figures and Tables

**Table 1:** Socio-demographic characteristic of the study group.

	*n* Respondents	% Respondents
Marital status		
Single	2	4.1
Married	36	73.5
Divorced	2	4.1
Widow	8	16.3
Informal relationship	1	2.0
Education		
Primary	12	24.5
Professional	15	30.6
Secondary	17	34.7
Higher	5	10.2
Distance from home to cancer center		
Up to 5 km	3	6.1
6–15 m	0	0.0
16–30 km	2	4.1
31–45 km	4	8.2
Over 45 km	40	81.6
Source of income		
Work	15	30.6
Benefit	13	26.5
Pension	8	16.3
Welfare	3	6.1
Other	10	20.4

**Table 2:** Clinical characteristics of the study group.

	*n* Respondents	% Respondents
Histology		
Planoepitheliale	44	89.8
Infiltratio carcinomatosa	5	10.2
Stage		
IB	4	8.2
IIA	7	14.3
IIB	4	8.2
IIIB	25	51.0
IVA	1	2.0
No data	8	16.3
No. of radiotherapy sessions		
22–24	12	24.5
25–27	31	63.3
28–32	6	12.2
Irradiation dose (Gy)		
44–46	10	20.4
48–50	21	42.8
52–56	18	35.8
Chemotherapy		

No	14	28.6
Yes	35	71.4
Surgical cancer treatment		
No	36	73.5
Yes	13	26.5
	49	100%

**Table 3:** Quality of life (QoL) and overall health assessment by the respondents.

Variable	Stage of Study	N Valid	Mean	SD	Min	Max	Lower Quartile	Upper Quartile	Quartile Interval	Sć Skew	Standard Skew Error
Overall health	I	49	4.53	1.54	1.00000	7.0000	4.00000	5.0000	1.00000	−0.14864	0.339828
	II	49	4.71	1.29	2.00000	7.0000	4.00000	6.0000	2.00000	0.32014	0.339828
	III	49	4.71	1.46	1.00000	7.0000	4.00000	6.0000	2.00000	−0.19232	0.339828
	IV	49	4.43	1.68	1.00000	7.0000	3.00000	6.0000	3.00000	−0.00781	0.339828
Quality of life	I	49	4.76	1.55	1.00000	7.0000	4.00000	6.0000	2.00000	−0.27357	0.339828
	II	49	4.76	1.28	2.00000	7.0000	4.00000	6.0000	2.00000	0.11102	0.339828
	III	49	4.78	1.52	1.00000	7.0000	4.00000	6.0000	2.00000	−0.83295	0.339828
	IV	49	4.51	1.71	1.00000	7.0000	3.00000	6.0000	3.00000	−0.14860	0.339828

SD: Standard Deviation, Min: Minimum, Max: Maximum.

**Table 4:** Overall health and quality of life assessment by the respondents.

	Before Treatment					Directly after Treatment					5–6 Months after Treatment					5 Years after Treatment				
	Mean	Median	SD	Min.	Max	Mean	Median	SD	Min.	Max	Mean	Median	SD	Min.	Max	Mean	Median	SD	Min.	Max
Global health status/ QoL (standardized)	60.71	66.67	25.17	0.00	100.00	62.24	58.33	20.91	25.00	100.00	62.41	66.67	23.58	0.00	100.00	57.82	50.00	26.97	0.00	100.00

M: Median; SD: Standard Deviation; Min: Minimum; Max: Maximum.

**Table 5:** Mean functional subscale values with rank determination.

	before Treatment					Rank	Directly after Treatment					Rank	5–6 Months after Treatment					Rank	5 Years after Treatment					Rank
	Mean	Median	SD	Min.	Max		Mean	Median	SD	Min.	Max		Mean	Median	SD	Min.	Max		Mean	Median	SD	Min.	Max	
Physical functioning (PF)	76.33	80.00	18.16	26.67	100.00	1	67.48	73.33	18.74	26.67	100.00	2	67.35	73.33	21.74	6.67	100.00	4	62.86	66.67	22.73	20.00	100.00	4
Role functioning (RF)	75.85	83.33	27.23	0.00	100.00	2	56.46	50.00	30.01	0.00	100.00	5	73.81	83.33	29.66	0.00	100.00	2	69.73	66.67	28.60	0.00	100.00	2
Emotional functioning (EF)	52.38	58.33	26.02	0.00	100.00	5	60.37	66.67	23.73	0.00	100.00	4	59.69	66.67	21.41	8.33	100.00	5	54.08	58.33	27.17	0.00	100.00	5
Memory and concentration (CF)	71.09	66.67	26.08	0.00	100.00	4	68.71	66.67	25.83	0.00	100.00	1	68.37	66.67	28.10	0.00	100.00	3	68.37	66.67	26.84	0.00	100.00	3
Social functioning (SF)	73.47	83.33	31.17	0.00	100.00	3	67.01	66.67	28.77	0.00	100.00	3	80.61	83.33	23.41	0.00	100.00	1	73.81	66.67	24.30	0.00	100.00	1
Financial impact of the disease (FI)	44.90	33.33	32.31	0.00	100.00	6	46.94	33.33	30.37	0.00	100.00	6	46.94	33.33	27.99	0.00	100.00	6	41.50	33.33	36.98	0.00	100.00	6

M: Median; SD: Standard Deviation; Min: Minimum; Max: Maximum.

**Table 6:** Mean somatic subscale values with rank determination.

	before Treatment					Rank	Directly after Treatment					Rank	5–6 Months after Treatment					Rank	5 Years after Treatment					Rank
	Mean	Median	SD	Min.	Max		Mean	Median	SD	Min.	Max		Mean	Median	SD	Min.	Max		Mean	Median	SD	Min.	Max	
Fatigue (FA)	37.87	3333	23.34	0.00	100.00	2	43.54	33.33	23.88	0.00	100.00	2	45.35	33.33	22.32	0.00	100.00	1	48.07	44.44	22.84	0.00	100.00	2
Nausea and vomiting (NV)	7.48	0.00	12.30	0.00	50.00	8	21.43	16.67	23.32	0.00	83.33	7	9.52	0.00	14.03	0.00	50.00	8	13.27	0.00	18.94	0.00	66.67	7
Pain (PA)	29.25	33.33	25.80	0.00	100.00	3	29.93	33.33	24.76	0.00	83.33	5	30.61	33.33	24.14	0.00	100.00	3	37.76	33.33	29.22	0.00	100.00	3
Dyspnea (DY)	14.29	0.00	21.52	0.00	100.00	6	18.37	0.00	23.63	0.00	100.00	8	23.81	0.00	28.87	0.00	100.00	4	27.21	33.33	27.78	0.00	100.00	5
Insomnia (SL)	44.22	33.33	31.47	0.00	100.00	1	46.94	33.33	32.57	0.00	100.00	1	42.18	33.33	31.01	0.00	100.00	2	51.70	33.33	35.40	0.00	100.00	1
Loss of appetite (AP)	16.33	0.00	26.46	0.00	100.00	5	43.54	33.33	30.58	0.00	100.00	2	17.69	0.00	21.63	0.00	100.00	6	23.81	33.33	28.05	0.00	100.00	6
Constipation (CO)	28.57	33.33	29.66	0.00	100.00	4	26.53	33.33	25.44	0.00	100.00	6	15.65	0.00	22.67	0.00	100.00	7	21.09	0.00	29.42	0.00	100.00	8
Diarrhea (DI)	11.56	0.00	17.42	0.00	66.67	7	31.97	33.33	28.02	0.00	100.00	4	23.13	33.33	23.77	0.00	66.67	5	37.41	33.33	32.37	0.00	100.00	4

M: Median; SD: Standard Deviation; Min: Minimum; Max: Maximum.

**Table 7:** Differences in QOL (Quality of Life) scores in different time periods.

	Stage I		Stage II		Stage III		Stage IV		ANOVA with Secondary Measurement			
	M	sd	M	sd	M	sd	M	sd	F	df	*p*	η^2^
**PF**	76.33	18.16	67.48	18.74	67.35	21.74	62.86	22.73	8.29	3.144	0.000 ***	0.147
**RF**	75.85	27.23	56.46	30.01	73.81	29.66	69.73	28.60	6.73	3.144	0.000 ***	0.123
**EF**	52.38	26.02	60.37	23.73	59.69	21.41	54.08	27.17	2.75	3.144	0.045 *	0.054
**CF**	71.09	26.08	68.71	25.83	68.37	28.10	68.37	26.84	0.30	3.144	0.826	0.006
**SF**	73.47	31.17	67.01	28.77	80.61	23.41	73.81	24.30	2.72	3.144	0.046 *	0.054
**FA ^a^**	37.87	23.34	43.54	23.88	45.35	22.32	48.07	22.84	3.39	3.123	0.026 *	0.066
**NV ^a^**	7.48	12.30	21.43	23.32	9.52	14.03	13.27	18.94	8.83	2.111	0.000 ***	0.155
**PA**	29.25	25.80	29.93	24.76	30.61	24.14	37.76	29.22	1.72	3.144	0.165	0.035
**DY**	14.29	21.52	18.37	23.63	23.81	28.87	27.21	27.78	4.97	3.144	0.003 **	0.094
**SL**	44.22	31.47	46.94	32.57	42.18	31.01	51.70	35.40	1.25	3.144	0.294	0.025
**AP**	16.33	26.46	43.54	30.58	17.69	21.63	23.81	28.05	15.93	3.144	0.000 ***	0.249
**CO ^a^**	28.57	29.66	26.53	25.44	15.65	22.67	21.09	29.42	3.36	3.122	0.027 *	0.065
**DI ^a^**	11.56	17.42	31.97	28.02	23.13	23.77	37.41	32.37	11.67	3.121	0.000 ***	0.196
**FI**	44.90	32.31	46.94	30.37	46.94	27.99	41.50	36.98	0.53	3.144	0.664	0.011

* p ≤ 0.05; ** p ≤ 0.01; *** p ≤ 0.001; ^a^—due to nonfulfillment of the sphericity assumption Greenhouse–Geisser correction has been applied. Sd—Standard Deviation, M—Mean, F—variation between sample means, df—degrees of freedom, η^2^—an effect size measure for one-way or factorial.

**Table 8:** Comparison of QOL (Quality of Life) scores between stages based on estimated marginal mean values with the application of Bonferroni correction for multiple comparisons (levels of significance from Table 7).

	SI-SII	SI-SIII	SI-SIV	SII-SIII	SII-SIV	SIII-SIV
**PF**	0.003 **	0.013 *	0.000 ***	0.999	0.844	0.594
**RF**	0.001 ***	0.999	0.999	0.004 **	0.087	0.999
**EF**	0.166	0.146	0.999	0.999	0.748	0.431
**SF**	0.999	0.780	0.999	0.067	0.999	0.490
**FA**	0.666	0.327	0.060	0.999	0.944	0.999
**NV**	0.001 ***	0.999	0.184	0.003 **	0.153	0.882
**DY**	0.999	0.071	0.005 **	0.618	0.132	0.999
**AP**	0.000 ***	0.999	0.656	0.000 ***	0.003 **	0.709
**CO**	0.999	0.071	0.882	0.116	0.999	0.618
**DI**	0.000 ***	0.004 **	0.000 ***	0.342	0.999	0.053

* p ≤ 0.05; ** p ≤ 0.01; *** p ≤ 0.001. Physical functioning (PF); Role functioning (RF); Emotional functioning (EF); Social functioning (SF); Fatigue (FA); Nausea and vomiting (NV); Dyspnea (DY); Loss of appetite (AP); Constipation (CO); Diarrhea (DI).

**Table 9:** Mean levels of anxiety and depression among respondents at different stages of the study.

Statistics	Before Treatment		Directly after Treatment		5–6 Months after Treatment		5 Years after Treatment	
	Anxiety	Depression	Anxiety	Depression	Anxiety	Depression	Anxiety	Depression
Mean	8.49	5.76	7.35	5.45	7.78	5.02	8.12	5.69
Median	8	5	7	5	7	4	8	6
SD	4.84	440	4.01	3.83	4.55	4.08	4.65	4.31
Min	0	0	0	0	0	0	0	0
Max	19	19	16	13	18	13	18	14

SD: Standard Deviation, Min: Minimum, Max: Maximum.

## Data Availability

The data presented in this study are available on request from the corresponding author.
